# Identification of Endosymbiotic Virus in Small Extracellular Vesicles Derived from *Trichomonas vaginalis*

**DOI:** 10.3390/genes13030531

**Published:** 2022-03-17

**Authors:** Seow-Chin Ong, Wei-Hung Cheng, Fu-Man Ku, Chih-Yu Tsai, Po-Jung Huang, Chi-Ching Lee, Yuan-Ming Yeh, Petr Rada, Ivan Hrdý, Ravi Kumar Narayanasamy, Tamara Smutná, Rose Lin, Hong-Wei Luo, Cheng-Hsun Chiu, Jan Tachezy, Petrus Tang

**Affiliations:** 1 Department of Parasitology, College of Medicine, Chang Gung University, Taoyuan 333, Taiwan; seowchin.ong@gmail.com (S.-C.O.); mann731025@mail.cgu.edu.tw (F.-M.K.); winnie255137@gmail.com (C.-Y.T.); lavender@mail.cgu.edu.tw (R.L.); maple890817@gmail.com (H.-W.L.); 2 Department of Medical Laboratory Science, College of Medical Science and Technology, I-Shou University, Kaohsiung 824, Taiwan; whcheng@isu.edu.tw; 3 Department of Biomedical Sciences, College of Medicine, Chang Gung University, Guishan District, Taoyuan 333, Taiwan; pjhuang@gap.cgu.edu.tw; 4 Genomic Medicine Core Laboratory, Chang Gung Memorial Hospital, Linkou Branch, Taoyuan 333, Taiwan; chichinglee@cgu.edu.tw (C.-C.L.); ymyeh@cgmh.org.tw (Y.-M.Y.); 5 Department of Computer Science and Information Engineering, College of Engineering, Chang Gung University, Taoyuan 333, Taiwan; 6 Department of Parasitology, Faculty of Science, Biotechnology and Biomedicine Centre of the Academy of Sciences and Charles University in Vestec (BIOCEV), Průmyslová 595, 252 42 Vestec, Czech Republic; radapetr81@gmail.com (P.R.); ivan.hrdy@natur.cuni.cz (I.H.); stallionravi27@gmail.com (R.K.N.); tamara.smutna@natur.cuni.cz (T.S.); 7 Molecular Infectious Disease Research Center, Chang Gung Memorial Hospital, Linkou Branch, Taoyuan 333, Taiwan; chchiu@adm.cgmh.org.tw

**Keywords:** *Trichomonas vaginalis*, *Trichomonasvirus*, extracellular vesicles, proteomics, New-Generation Sequencing

## Abstract

Accumulated evidence suggests that the endosymbiotic *Trichomonasvirus* (TVV) may play a role in the pathogenesis and drug susceptibility of *Trichomonas vaginalis*. Several reports have shown that extracellular vesicles (EVs) released from TVV-positive (TVV+) trichomonads can modulate the immune response in human vaginal epithelial cells and animal models. These results prompted us to examine whether EVs released from TVV+ isolates contained TVV. We isolated small extracellular vesicles (sEVs) from six *T. vaginalis* isolates that were either TVV free (ATCC 50143), harbored a single (ATCC 30236, ATCC 30238, T1), two (ATCC PRA-98), or three TVV subspecies (ATCC 50148). The presence of TVV subspecies in the six isolates was observed using reverse transcription-polymerase chain reaction (RT-PCR). Transmission electron microscopy (TEM) confirmed the presence of cup-shaped sEVs with a size range from 30–150 nm. *Trichomonas vaginalis* tetraspanin (TvTSP1; TVAG_019180), the classical exosome marker, was identified in all the sEV preparations. Liquid chromatography-tandem mass spectrometry (LC-MS/MS) analysis showed that all the sEVs isolated from TVV+ isolates contain viral capsid proteins derived from the same TVV subspecies in that isolate as demonstrated by RT-PCR. To provide more comprehensive information on the TVV subspecies population in other *T. vaginalis* isolates, we investigated the distribution of TVV subspecies in twenty-four isolates by mining the New-Generation Sequencing (NGS) RNAseq datasets. Our results should be beneficial for future studies investigating the role of TVV on the pathogenicity of *T. vaginalis* and the possible transmission of virus subspecies among different isolates via sEVs.

## 1. Introduction

*Trichomonas vaginalis* is an anaerobic flagellated protozoan that causes trichomoniasis, one of the most common sexually transmitted diseases worldwide. Approximately 156 million infections were reported worldwide in 2020 [1]. Infection is more likely to affect females [2,3]. However, only about 30% of infected patients develop clinical symptoms. Although most infections are asymptomatic, severe vaginal inflammation and adverse effects on pregnancy have been reported in female patients. The disease also affects the vagina, cervix, urethra, or a combination of the urogenital tracts [4,5].

*Trichomonas vaginalis* was the first reported parasite capable of altering host cells’ physical and biochemical properties via extracellular vesicles (EVs) or exosomes [6]. Exosomes are small extracellular vesicles (sEVs) with a diameter of 30–150 nm enveloped in a lipid bilayer membrane. Exosomes can transfer DNA, RNA, proteins, and metabolites from the cell of origin to other cells, thereby altering the function of the target cell [7,8]. Pioneer studies on *T. vaginalis* exosomes revealed that exosomes isolated from the parasite also contain the classical exosomal marker tetraspanins (TSPs) [6]. TSPs are proteins consisting of four transmembrane domains termed tetraspanin-enriched microdomains (TEMs). These proteins play a role in many aspects of cell biology and physiology in coordinating intracellular and intercellular processes [9,10,11]. In vitro studies demonstrated that trichomonad exosomes can deliver protein or RNA to host cells and modulate the host immune system and host–parasite interactions. Olmos-Ortis et al. [12] suggested that exosome-like vesicles from *T. vaginalis* could favor the persistence of the parasite by reducing the inflammatory host response, have an immunomodulatory role on the cytokine profile induced by the parasite, and promote a decrease in the inflammatory process in mice infected with *T. vaginalis.*

*Trichomonasvirus* (TVV), is a double-stranded RNA (dsRNA) virus of the Totiviridae family and the first virus identified from a protozoan parasite. [13,14]. The TVV has a nonsegmented genome of 4.5 to 5 kbp that contains two overlapping open reading frames for the capsid protein and RNA-dependent RNA polymerase (RdRp). Four different TVV subspecies (TVV1, TVV2, TVV3, and TVV4) were identified based on phylogenetic analyses and comparisons of genomic sequences [15]. Although TVV can only be transmitted vertically, a single cell can be infected by a single or up to four TVV subspecies in some isolates [16]. The TVV infection rate among *T. vaginalis* strains from different geographic origins ranges from 40 to 100%, with TVV1 being the dominant detected subspecies in the USA [17] and Netherlands [18].

TVV is known to alter the expression profile of host cysteine proteases, a known virulence factor for species involved in the degradation of human immune proteins [19]. This viral endosymbiont seems to influence and modulate protozoan virulence [20]. Cysteine proteases promote *T. vaginalis* cytoadhesion to the vaginal epithelium and mediate cytotoxicity, which aids in the survival of the parasite and host immune evasion [21,22]. In addition, they also increased the expression of a prominent surface immunogen named P270 [20,23,24]. Thus, TVV-positive *T. vaginalis* isolates can express P270 in the cytoplasm and cell surface. Moreover, Govender et al. [25] demonstrated that viral endosymbiosis altered the immunomodulatory properties of sEVs spreading from the site of infection to non-infected immune effector cells. More specifically, when human vaginal epithelial cells were incubated with *T. vaginalis* isolates with TVV-positive and TVV-negative, sEVs from TVV-negative but not TVV-positive parasites cultured alone caused NF-ĸB activation and an increase in IL-8 and RANTES expression, which provide innate immune defense at the gate to the upper reproductive tract [25].

To the best of our knowledge, the role of *T. vaginalis* viral endosymbiosis in sEVs is still unknown. Our report demonstrates for the first time that TVV could exist in sEVs released from *T. vaginalis*. We also used RNAseq data generated by our team or available in the public domain to identify TVVs in twenty-four isolates. Our study highlighted the possible role of exosomal TVV on the transmission of TVV among different isolates and the effect of TVV subspecies on the pathogenicity of trichomonal vaginitis.

## 2. Materials and Methods

### 2.1. Trichomonas vaginalis Culture

*Trichomonas vaginalis* ATCC 30236, 30238, 50148, 50143, PRA-98 and T1 isolates were cultured at 37 °C in yeast extract and iron-serum (YI-S) medium containing 80 μM ferrous ammonium citrate (Sigma-Aldrich, St. Louis, MO, USA) [26]. The cells for assays were harvested at the mid-log phase. The trypan blue exclusion assay was used to count the number of viable cells.

### 2.2. Detection of TVV through Reverse Transcription-Polymerase Chain Reaction (RT-PCR)

Total RNA from the logarithmic phase cultures of the six *T. vaginalis* isolates was extracted with the GENEzol™ TriRNA Pure Kit (Geneaid, New Taipei City, Taiwan) following the manufacturer’s protocol. Then, the first-strand cDNA was reverse-transcribed from the total RNA extract using the Superscript™ III First-Strand Synthesis System (Invitrogen; Life Technologies, Carlsbad, CA, USA). Subsequently, the double-stranded DNA of the target virus genes was amplified using species-specific primers and 2 × Super Hi-Fi Taq PCR MasterMix with loading dye (Biotools, New Taipei City, Taiwan). The species-specific primer pairs used included TVV1F2875-TVV1R3443, TVV2F2461-TVV2, TVV3F61-TVV3R482, and TVV4F1338-TVV4R1834, which were designed by Goodman et al. [13]. Each of the reaction mixtures (25 µL) contained 10 µM of each primer, 12.5 mL of 2 × Super Hi-Fi Taq PCR MasterMix with loading dye, and 1 µg of template from reverse transcription, according to the manufacturer’s protocol (Biotools, New Taipei City, Taiwan). The thermal cycling parameters were as follows: inactivation of reverse transcriptase and initial PCR activation at 94 °C for 3 min; 30 cycles of 94 °C for 30 sec, 55 °C for 30 sec and 72 °C for 1 min; and a final extension step of 72 °C for 5 min. Finally, the reaction remained at 4 °C. Amplified cDNA fragments were visualized after separation in a 1.5% agarose gel in Tris-Acetate-EDTA (TAE) buffer containing FluoroVue™ Nucleic Acid Safe stain (SMOBIO Technology Inc., Taipei, Taiwan).

### 2.3. The Isolation of Small Extracellular Vesicles

The sEVs were isolated following the method from Gupta et al. [27] with some modifications. One liter of each *T. vaginalis* culture at a density of approximately 2 × 10^6^ cells/mL was harvested by centrifugation and washed three times with 1 × PBS (pH 6). The washed cells were then suspended in YIS media without serum and cultured for approximately 4 h at 37 °C. Cells were removed by centrifugation at 2000× *g* for 15 min followed by centrifugation at 10,000× *g* for 30 min. The cell-free supernatant was filtered through a 0.22 µm filter and concentrated using 10 kDa MWCO mPES hollow fiber (Lefo Science Co., Ltd, Taipei, Taiwan) to approximately 10 mL. The filtered cell-free media were further concentrated using an Amicon Ultra15 centrifugal filter unit (MWCO 10 kDa; Millipore Corporation, Bedford, MA, USA) to a final volume of approximately 3.5 mL. The concentrated cell-free medium was loaded slowly over 0.5 mL of 30% sucrose solution and ultracentrifuged at 100,000× *g* for 2 h at 4 °C using a swinging bucket rotor (P56ST; Hitachi Koki Co., Ltd., Minato, Tokyo, Japan). Subsequently, the supernatant was discarded. The sucrose layer (~0.6 mL) was resuspended in 3.4 mL of 1 × PBS (pH 7) and ultracentrifuged again at 100,000× *g* for 2 h at 4 °C to pellet down the sEVs. The isolated sEVs were resuspended in approximately 20 µL of 1 × PBS buffer (pH 7) and stored at −20 °C for LC-MS/MS analysis. The protein concentration of each sEV sample was detected using Bio-Rad Protein Assay Dye Reagent Concentrate.

### 2.4. Transmission Electron Microscopy (TEM)

An aliquot (3 µL) from each of the sEV solutions was coated onto a formvar carbon grid. Briefly, the sEV samples were fixed with 1% uranyl acetate. Then, the grids were air-dried and visualized using a JEM-2100 PLUS TEM (JEOL, Tokyo, Japan) at 100 kV.

### 2.5. Proteomics Data Analysis

For protein identification, 5 µg of each protein from each fraction was sent to the Molecular Medicine Research Center, Chang Gung University for proteomics analysis. As previously described, the proteins were subjected to in-solution tryptic digestion before LC-MS/MS analysis [28]. Each peptide sample was reconstituted with 0.1% formic acid (FA) and then analyzed by a nano-LC-LTQ-Orbitrap Hybrid Mass Spectrometer (Thermo Fisher, San Jose, CA, USA), as previously described [29]. Briefly, the sample was loaded across a trap column (Zorbax 300SB-C18, 0.3 × 5 mm; Agilent Technologies, Wilmington, DE, USA) at a flow rate of 0.2 μL/min in HPLC buffer (0.1% FA) and separated on a resolving 10 cm analytical C18 column (inner diameter, 75 μm) using a 15-μm tip (New Objective, Woburn, MA, USA). For database searching, mass spectrometry raw data files were analyzed by Proteome Discoverer Software (version 1.4.1.14; Thermo Fisher, San Jose, CA, USA), searched against the TrichDB protein database and TVV reference sequences using the Mascot search engine (Matrix Science, London, UK; version 2.5).

### 2.6. Identification of TVV Fragments by RNAseq and New-Generation Sequencing (NGS)

The total RNA of twenty-four isolates (CDC085; NYCB20; ATCC 50143; T1; ATCC 30236; B7268; NYCG31; TO16; ATCC 30001; ATCC 30238; GOR69; NYCA04; NYCE32_8; NYCF20; SD2; LSU180; CDC1103; CDC1123; CDC1132; ATCC PRA-98; NYH286; NYCC37; ATCC 50148; NYCD15) extracted using TRIzol reagent (Ambion; Life Technologies, Carlsbad, CA, USA) was converted into a library of cDNA fragments and sequenced using the Illumina HiSeqTM 2000. Approximately 60 million paired-end reads of 100–150 nt were generated from each cDNA library. The raw reads were processed using the in-house Comparative RNA-Sequencing Analysis Pipeline (CRSAP) installed in the Chang Gung Bioinformatics Center. Quality reads were mapped against the ‘ATCC PRA-98’ reference genome (TrichDB, release-50) and TVV reference sequences using the recommended default parameters. Reads per kilobase per million mapped reads (RPKM) of each gene was calculated and used as the normalized gene expression level. In addition to the RNAseq datasets generated in the present study, we also downloaded available *T. vaginalis* RNAseq datasets from the public domain to detect TVV fragments.

## 3. Results

### 3.1. Trichomonasvirus Detection by RT-PCR

**Figure 1:**
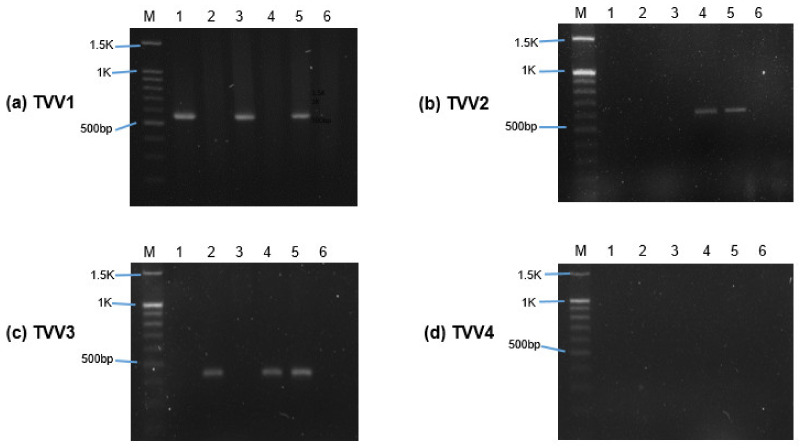
Detection of *Trichomonasvirus* (TVV) subspecies in *T. vaginalis* isolates using RT-PCR. (**a**–**d**) showed the separation of RT-PCR products amplified by TVV1, TVV2, TVV3, and TVV4 subspecies-specific primers on 1.5% agarose gel. M indicates the marker (DNA ladder). Lanes labeled 1 to 6 represent *T. vaginalis* isolates ATCC 30236, ATCC 30238, T1, ATCC PRA-98, ATCC 50148, and ATCC 50143.

Among the six isolates of *T. vaginalis* used for sEV isolation, five isolates were positive for at least one subspecies of TVV. Only one isolate (ATCC 50143) was negative for all the virus subspecies (Figure 1). A specific band confirmed the presence of TVV on the agarose gel at approximately 569 bp (TVV1), 625 bp (TVV2), 437 bp (TVV3), and 514 bp (TVV4), respectively. Isolates of ATCC 30236 and T1 were positive only for TVV1, while ATCC 30238 only harbored TVV3. Isolates of ATCC 50148 showed co-infection with TVV1, TVV2, and TVV3, whereas ATCC PRA-98 was co-infected with TVV2 and TVV3. The most prevalent viruses were TVV1 (isolates of ATCC 30236, T1, and ATCC 50148) and TVV3 (isolates of ATCC 30238, PRA-98, and 50148).

### 3.2. Morphology of T. vaginalis Small Extracellular Vesicles

**Figure 2:**
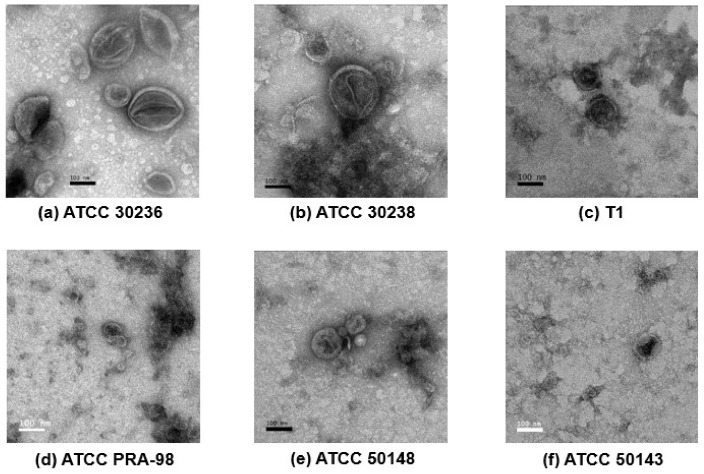
Transmission electron microscopy (TEM) of the small extracellular vesicles (sEVs) derived from *T. vaginalis* isolates; (**a**) ATCC 30236, (**b**) ATCC 30238, (**c**) T1, (**d**) ATCC PRA-98, (**e**) ATCC 50148 and (**f**) ATCC 50143. The morphology of purified sEVs of the six *T. vaginalis* isolates after ultracentrifugation were identified using negative staining with 1% uranyl acetate and observed under TEM. (Original magnification, 80,000×; scale bar = 100 nm).

TEM confirmed the presence of sEVs in all the *T. vaginalis* preparations. Overall, all the sEVs isolated from different isolates showed a cup shape with bilayer membranes, approximately 30–150 nm in diameter (Figure 2), except for isolate ATCC PRA-98, in which the sEVs seemed smaller than the other isolates. The larger particle sizes of sEVs from isolate ATCC-PRA 98 were less than 100 nm. In addition, virus-like particles (VLPs) with a diameter between 30 to 40 nm were found in the sEVs preparation as shown in Supplementary Figure S1.

### 3.3. Proteomics Analysis of Trichomonasvirus and Tetraspanin by LC-MS/MS

To clarify the existence of TVV in the sEVs released by *T. vaginalis*, we used a proteomics approach to detect viral proteins in sEVs. As shown in Table 1, an LC-MS/MS analysis confirmed the presence or absence of the TVV capsid protein in all the sEV proteome. The genotypes of the TVV1, TVV2, and TVV3 capsid proteins detected by LC-MS/MS analysis were identical to those of the TVV genotypes detected by RT-PCR. The only exception is the genotype TVV2, which was detected only in the sEVs from ATCC 50148 but not in the sEVs of ATCC PRA-98, as detected by RT-PCR. A possible cause is that the concentration of viral protein in the sEV sample of ATCC PRA-98 is below the limit of detection by LC-MS/MS. A detailed description and the peptide sequences of the TVV capsid proteins from the sEVs are shown in Supplementary Table S1.

As shown in Table 2, LC-MS/MS identified the exosome marker tetraspanin TvTSP1 (Accession no.: TVAG_019180) in all the isolates. In addition to TvTSP1, four isolates, ATCC 30238, 50148, 50143, and T1, also expressed TvTSP8 (Accession no.: TVAG_008950), while TvTSP6 (Accession no.: TVAG _460770) was only expressed in ATCC 30236 and 50143. A detailed description and the peptide sequences of tetraspanin proteins from the sEVs are shown in Supplementary Table S2.

### 3.4. Identification of TVV Fragments by RNAseq and NGS

Table 3 shows an analysis of twenty-four *T. vaginalis* RNA sequencing datasets generated from the present study or downloaded from the public domain. TVV subspecies-specific reads in each dataset were identified by mapping to the TVV reference sequences. Among the 24 isolates studied, 18 (75%) isolates were infected with TVV1, followed by 11 (46%) isolates with TVV3, six (25%) isolates with TVV2, and five (21%) isolates with TVV4. Only five isolates (21%) were detected with TVV1 alone, and two isolates (8.3%) were detected with TVV3 alone. This result is identical to the TVV subspecies identified by the sEV proteome and RT-PCR from ATCC 30236 and ATCC 30238. The ATCC-GOR69 isolate was co-infected with TVV1 and TVV2, while the ATCC PRA-98 isolate was co-infected with TVV2 and TVV3. In addition, four isolates were co-infected with TVV1 and TVV3, and another four isolates were co-infected with TVV1 and TVV4. Furthermore, three isolates were co-infected with TVV subspecies 1, 2 and 3, including isolate ATCC 50148, which showed the same results as in RT-PCR agarose gel and LC-MS/MS proteomics data. Finally, only one isolate (NYCD15) was co-infected with all four subspecies of TVVs.

## 4. Discussion

Our study demonstrated that TVV could exist in sEVs released from the host cell for the first time. More importantly, our proteomics analysis showed that the presence of TVV in sEVs isolated from the six *T. vaginalis* isolates was similar to the results of RT-PCR and NGS data of the TVV subspecies detected in each cell of the *T. vaginalis* isolates. This result demonstrated that TVV can be packed into *T. vaginalis*, and we speculated that sEVs-mediated transmission might be a possible mechanism for TVV infection in TVV-free *T. vaginalis*.

In this study, we used TEM and LC-MS/MS as tools to confirm the size of the isolated sEVs and screen for the classical exosome marker TSP. TEM confirmed the presence of sEVs in the size range of exosome-like vesicles as defined in the previous literatures. This study identified the classical marker TSP in all of the sEV proteomes. Tetraspanin TvTSP1 was identified in all the sEV isolates, while TvTSP8 and TvTSP6 were identified in four and two isolates, respectively. Twu et al. [6] and Nievas et al. [30] suggested that tetraspanin TvTSP1 and TvTSP8 proteins might be used as markers for EVs in *T. vaginalis* due to the presence of both TSPs in the proteome as of the EVs. Interestingly, TvTSP1 was upregulated in EVs upon exposure of the parasite to vaginal epithelial cells, suggesting that TvTSP1 may be involved in the host–parasite interaction [6,30]. Moreover, TvTSP1 and TvTSP6 were found to reside strongly on the surface (plasma membrane and flagella) and on the membranes of intracellular vesicles internally [6,31,32]. In addition, TvTSP8 was shown to be involved in parasite aggregation, suggesting a role for this protein in parasite:parasite communication. Further study on the mechanisms of the sEV function in host–parasite interactions should be undertaken to clarify the biological roles of sEVs.

Although there are many studies on viral endosymbiosis in various protozoan parasites such as *Trichomonas* [17,19], *Giardia* [33], *Leishmania* [34,35], *Plasmodium* [36] and *Crytosporidium* [37,38], only a few reports focus on the importance of sEVs in viral-protozoan symbiosis. Atayde et al. [39] reported that *Leishmania* RNA Virus 1 (LLRV1) enveloped within exosomes could exit from *Leishmania* to enhance virus transmission.

In *T. vaginalis*, TVV is non-infectious to the human and animal hosts due to the absence of virion-associated machinery that allows entrance into the cells. TVV in infected *T. vaginalis* strains is retained by TVV segregation during cytokinesis and can only be transferred to daughter cells during binary fission. Co-incubation of purified TVV viral particle with the parasite showed that *T. vaginalis* trophozoite could engulf the virus by endocytosis. Attempts to establish a stable TVV infection in TVV-free *T. vaginalis* strains in vitro have been unsuccessful so far [14,40]. Thus, TVV transmission between the parasites remains a mystery.

Several studies have proposed that sEVs released from TVV-infected isolates could modulate the host immune response. *T. vaginalis* sEVs were shown to deliver their soluble content and transfer lipids when the membrane of the sEVs fused with the membrane of the host cells [6,41]. Twu et al. [6] suggested that in addition to the protein cargo, a yet uncharacterized small RNA ranging in size from 25 to 200 nt packaged in *T. vaginalis* sEVs may play a role in parasite–parasite or host–parasite interactions. sEVs released by protozoan parasites could facilitate biological functions, including parasite adherence, differentiation, drug resistance, and tissue tropism [42,43]. Remarkably, Twu et al. also demonstrated that sEVs released by *T. vaginalis* could modulate the production of cytokines IL6 and IL8 and potentially prime host cells for parasite infection. Furthermore, sEVs from a highly adherent strain could enhance strong parasite attachment of a less adherent strain to epithelial cells [6].

Recently, Govender et al. reported that viral endosymbiosis could modify the immunomodulatory properties of sEVs. Mononuclear leukocytes increased their interleukin-6 (IL-6), IL-8, and tumor necrosis factor-α (TNF-α) output in response to sEVs from TVV-negative but not isogenic TVV-positive parasites. It is theoretically possible that sEVs may carry whole TVV virions or genomic dsRNA [25]. The TVV virions are at most 45 nm in diameter [40] and may fit into sEVs with a size of approximately 100 nm. Here, we demonstrate for the first time that TVV could be packaged in *T. vaginalis* sEVs. Our proteomics study has successfully identified TVV capsid proteins in sEVs released from *T. vaginalis*. Although Govender et al. [25] could not identify any proteins of TVV origin in sEVs by LC-MS/MS, their experimental data supported the idea that viral endosymbiont may use sEVs as a vehicle for intercellular communications and deliver proteins to suppress host immune activation.

Based on our analysis of NGS RNA sequencing datasets of twenty-four isolates, we found that most of *T. vaginalis* isolates were infected with TVV1, followed by TVV3 and TVV2; while the level of uninfected trichomonad cells was relatively rare. This result is similar to the report of Jehee et al. [18] in the Netherlands. In addition, Goodman et al. [17] in the USA, Masha et al. [44] in Kenya and da luz Becker et al. in Brazil [45] reported that the most prevalent virus in TVV-infected isolates was TVV1, followed by TVV2, TVV3, and TVV4. Rivera et al. [46] reported that TVV1 and TVV2 are the major TVV subspecies in the Philipines. However, Margarita et al. [47] reported that TVV2 was the dominant subspecies in Italian trichomoniasis, followed by TVV1, TVV3 and TVV4. Previous studies showed that *T. vaginalis* can be co-infected with more than one TVV subspecies, and these dsRNA viruses are transmitted vertically during *T. vaginalis* binary fission [16,17,18,44,45,46,47]. The presence of multiple TVV subspecies in a single *T. vaginalis* cell may complicate their roles in the upregulation of the inflammatory response and increase the severity of symptoms in patients with trichomoniasis [48,49]. Magarita et al. [47] reported that more than one TVV subspecies existed in 75% of positive trichomonad isolates. Our RT-PCR and proteomics results showed that two of the six isolates we studied were co-infected with TVV subspecies. Isolate ATCC PRA-98 had co-infected with TVV2 and TVV3, while ATCC 50148 had co-infected with TVV1, TVV2 and TVV3, whereas in our analysis of NGS RNA sequencing datasets of twenty-four isolates, 66.7% of positive isolates had co-infection with more than one TVV subspecies. Remarkably, Goodman et al. [17] found that TVV1 was evolutionarily closer to TVV2, while TVV3 was closer to TVV4.

## 5. Conclusions

In conclusion, our results suggested that TVV could be packaged in sEVs and may transmit to host cells via sEVs. Although experimental data from the present study confirmed the presence of TVV in sEVs released from *T. vaginalis*, the possible role of exosomal TVV on the transmission of TVV among different isolates and the effect of TVV subspecies on the pathogenicity of trichomonal vaginitis is still not clear. The major hurdle is that TVV is transmitted only vertically. A recent report demonstrated that cytidine nucleoside analog is an effective antiviral drug against TVV [50]. Reliable and reproducible methods to transfect the virus into infected cells or eliminate the virus from infected cells are still unavailable. Further studies should be performed to characterize the sEV content released from TVV+ or TVV− host cells and explore its effect on the pathogenicity of *T. vaginalis*.

## Figures and Tables

**Table 1:** The presence or absence of the *Trichomonasvirus* (TVV) subspecies in each proteome of small extracellular vesicles (sEVs) by LC-MS/MS.

Isolate	*Trichomonasvirus* Subspecies			
	TVV1	TVV2	TVV3	TVV4
ATCC 30236	+	−	−	−
ATCC 30238	−	−	+	−
T1	+	−	−	−
ATCC PRA-98	−	−	+	−
ATCC 50148	+	+	+	−
ATCC 50143	−	−	−	−

**Table 2:** Type of tetraspanin proteins present in each proteome of sEVs by LC-MS/MS.

Isolate	Type of Tetraspanin		
	TvTSP1	TvTSP6	TvTSP8
ATCC 30236	+	+	−
ATCC 30238	+	−	+
T1	+	−	+
ATCC PRA-98	+	−	−
ATCC 50148	+	−	+
ATCC 50143	+	+	+

**Table 3:** Identification of TVV subspecies by RNA sequencing and NGS.

Isolate	No. of TVV Subspecies	TVV1	TVV2	TVV3	TVV4
CDC085	0	−	−	−	−
NYCB20	0	−	−	−	−
ATCC 50143	0	−	−	−	−
T1	1	+	−	−	−
ATCC 30236	1	+	−	−	−
B7268	1	+	−	−	−
NYCG31	1	+	−	−	−
TO16	1	+	−	−	−
ATCC 30001	1	−	−	+	−
ATCC 30238	1	−	−	+	−
GOR69	2	+	+	−	−
NYCA04	2	+	−	+	−
NYCE32_8	2	+	−	+	−
NYCF20	2	+	−	+	−
SD2	2	+	−	+	−
LSU180	2	+	−	−	+
CDC1103	2	+	−	−	+
CDC1123	2	+	−	−	+
CDC1132	2	+	−	−	+
ATCC PRA-98	2	−	+	+	−
NYH286	3	+	+	+	−
NYCC37	3	+	+	+	−
ATCC 50148	3	+	+	+	−
NYCD15	4	+	+	+	+

## Data Availability

All data generated or analyzed during this study are included in the published article and its Supplementary Materials.

## Supplementary Materials

The following supporting information can be downloaded at: https://www.mdpi.com/article/10.3390/genes13030531/s1, Table S1: The details of different *Trichomonasvirus* capsid proteins in each proteome of sEVs identified by LC-MS/MS analysis; Table S2: The details of different tetraspanin proteins in each proteome of sEVs identified by LC-MS/MS analysis. Figure S1: TEM image of sEVs preparation in isolate ATCC 50148.
