# Supplementary material for: Identification of Endosymbiotic Virus in Small Extracellular Vesicles Derived from Trichomonas vaginalis
Source: Genes (Basel). 2022 Mar 17;13(3):531. doi: 10.3390/genes13030531 (PMC8951798; doi:10.3390/genes13030531)
Supplement: Supplementary file 1 [file genes-13-00531-s001.zip › Supplementary Figure S1.pptx]

## Slide 1
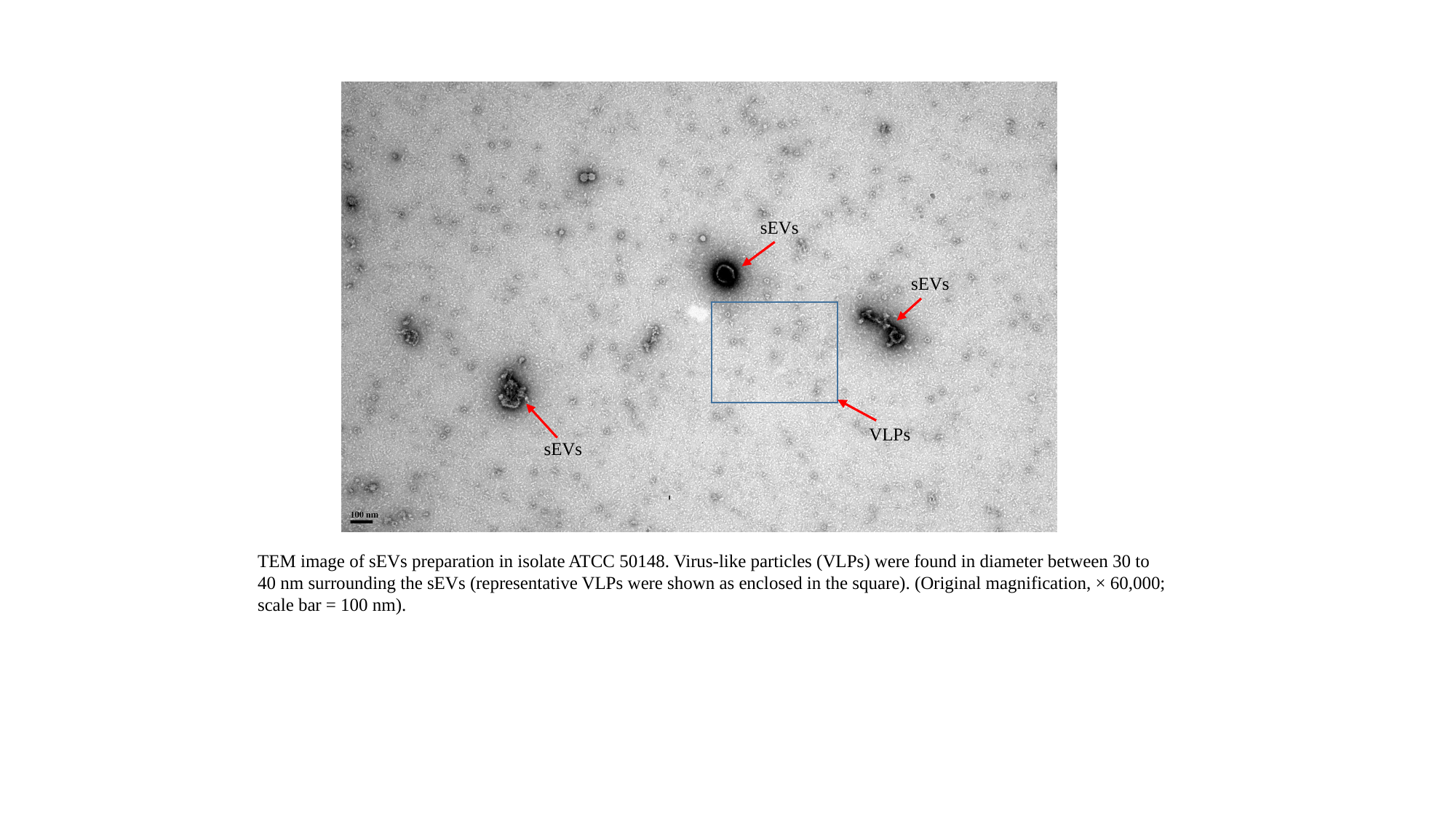

sEVs
sEVs
 VLPs
sEVs
TEM image of sEVs preparation in isolate ATCC 50148. Virus-like particles (VLPs) were found in diameter between 30 to 40 nm surrounding the sEVs (representative VLPs were shown as enclosed in the square). (Original magnification, × 60,000; scale bar = 100 nm).
